# Comparison of Prognostic Factors for Merkel Cell Carcinoma, Mucosal Melanoma and Cutaneous Malignant Melanoma: Insights into Their Etiologies

**DOI:** 10.3390/curroncol30040301

**Published:** 2023-03-31

**Authors:** Leslie K. Dennis, Heidi E. Brown, Amanda K. Arrington

**Affiliations:** 1 Department of Epidemiology and Biostatistics, Mel and Enid Zuckerman College of Public Health, University of Arizona, Tucson, AZ 85724, USA; heidibrown@arizona.edu; 2 Department of Surgery, Houston Methodist Hospital, Houston, TX 77030, USA; akarrington@houstonmethodist.org

**Keywords:** cutaneous melanoma, epidemiology, etiology, Merkel cell carcinoma, mucosal melanoma, SEER Program

## Abstract

Little is known about the epidemiology of Merkel cell carcinoma (MCC) and mucosal melanoma (MM). Using the United States (US) National Cancer Institute’s Surveillance, Epidemiology, and End Results (SEER) program data, we compared MCC and MM with cutaneous malignant melanoma (CMM) with respect to incidence rates and prognostic factors to better understand disease etiologies. We describe the proportional incidences of the three cancers along with their survival rates based on 20 years of national data. The incidence rates in 2000–2019 were 203.7 per 1,000,000 people for CMM, 5.9 per 1,000,000 people for MCC and 0.1 per 1,000,000 people for MM. The rates of these cancers increased over time, with the rate of MM tripling between 2000–2009 and 2010–2019. The incidences of these cancers increased with age and rates were highest among non-Hispanic Whites. Fewer MCCs and MMS were diagnosed at the local stage compared with CMM. The cases in the 22 SEER registries in California were not proportional to the 2020 population census but instead were higher than expected for CMM and MCC and lower than expected for MM. Conversely, MM rates were higher than expected in Texas and New York. These analyses highlight similarities in the incidence rates of CMM and MCC—and differences between them and MM rates—by state. Understanding more about MCC and MM is important because of their higher potential for late diagnosis and metastasis, which lead to poor survival.

## 1. Introduction

Many skin cancers are associated with ultraviolet radiation (UVR) through sun exposure and artificial tanning devices. Cutaneous malignant melanoma (CMM) and Merkel cell carcinoma (MCC) are categories of malignant skin cancers. CMMs are neoplasms that develop in melanocytes. Merkel cells are found in the top layer of the skin, close to the nerve endings that receive the sensation of touch. CMM and MCC do not have symptoms like non-skin cancers such as pain. MCC typically presents as a solitary, painless pink nodule on sun-exposed skin [1,2,3]. MCC is an aggressive skin cancer with high mortality [1,2,3,4,5,6]. Mucosal melanomas (MMs) arise in the mucosal epithelium and are very rare [7,8,9]. MMs show symptoms later in their progression, including bleeding and discoloration in most locations where they occur.

CMM etiology is fairly well described, with increased risk observed in fair-skinned individuals [10]. CMM is associated with intermittent sun exposure [11,12] such as sunburn during sunny vacations. The incidence of CMM in tanning bed users is also increasing, particularly among young women [13]. CMM occurrence is associated with the damage caused by intermittent sun exposure such as exposure of the skin to the sun during sunny vacations, leading to blistering sunburns. As CMM is associated with exposure to the sun and other UVR, its incidence increases with age.

Less is known about the etiology of MCC and MM. MCC was first described in 1972 [14,15,16]. It commonly occurs on sun-exposed body parts of fair-skinned individuals, similar to CMM [17]. UVR is therefore considered part of its etiology. The recently described Merkel Cell polyomavirus is believed to be a major cause of MCC [1,2,5,6,18], along with UVR-induced DNA damage [4,14,15,19,20]. MCC also occurs in immunocompromised individuals who are Merkel-Cell-polyomavirus negative [5,20], suggesting that multiple mechanisms are involved in its etiology.

MM occurs in the mucous membranes of the oral and nasal cavities, conjunctiva, genitourinary tract (especially the vulvar and vaginal area) and anorectal areas [7,21]. Several studies indicate later stage disease at the time of diagnosis, with poor prognosis and higher rates of metastasis [9,22,23]. MMs are so rare that a comprehensive analysis of risk factors has not been conducted. Additionally, very few studies have been conducted on the epidemiology of MM and most of our knowledge on MMs comes from case reports [7,21].

Despite their low incidence, the true impact of MCC and MM lies in their trend toward later diagnosis, higher rates of metastasis and overall poor survival. The purpose of this study was to describe the characteristics and rates of MCC and MM and to compare them with the more familiar CMM to better understand their etiologies. We described their proportional incidences and survival rates based on 20 years of national data from the United States (US).

## 2. Materials and Methods

### 2.1. Population

We examined MCC, MM and CMM cases in the National Cancer Institute’s Surveillance, Epidemiology, and End Results (SEER) program’s data in both SEER-17 and SEER-22 population-based US registries. Both registries were used because they contain different types of data. We analyzed cancer cases reported in the registries between 2000–2019. The SEER-17 registries include the Alaska Native tumor registry, and the California (San Francisco-Oakland, San Jose-Monterey, Los Angeles and greater California), Connecticut, Georgia (Atlanta, greater Georgia and rural Georgia), Hawaii, Iowa, Kentucky, Louisiana, New Mexico, New Jersey, Utah and the metropolitan area of Seattle-Puget Sound tumor registries [24]. The SEER-22 registries include those listed above as well as registries from Idaho, New York, Massachusetts, Texas and Illinois; however, they contain fewer risk factors for analysis. The SEER program also reports the 2020 census populations in each registry, which we verified for each state.

We restricted the analysis to malignant CMM cases as defined by the International Classification of Disease-Oncology (ICD-O) third edition behavior code for malignancy and primary site codes for skin—C44.0–C44.9 [25]. Analyses included all primary cases of malignant skin cancers based on the SEER definition of incidence, regardless of whether an individual had a previous cancer [24]. MCC cases were defined based on histology code 8247 (including oral cavity, digestive system, respiratory system, skin, breast, female genital system, male genital system, urinary system, and miscellaneous sites). MM cases were defined based on histology code 8746, with similar sites as MCC.

In 2022, SEER changed access to their data to be only through SEER*Stat (version 8.4.0.1). This restricted the data available to a limited number of factors, and SEER locations were not available when downloading some factors of interest for these analyses.

### 2.2. Statistical Methods

Descriptive statistics were used to describe the distribution of cases across prognostic factors. Rates were obtained through SEER*Stat [24]. Both case counts and rates are presented depending on the factor being described. We measured Breslow thickness to determine the t depth of the melanomas. Rates were age-adjusted for 5-year age-groups, except when explicitly reporting age. The standard for reporting cancer rates is per 100,000 population. However, we have reported rates per 1,000,000 population due to the low rates of MCC and MM. Trends for each cancer were calculated as annual percent change (APC) using Joinpoint Regression Program, version 4.9.1.0 [26,27].

Five-year survival was examined overall and across age groups and stage of disease, but data were only available for SEER-17. SEER*Stat states that the actuarial method was used with Ederer II method used for cumulative expected estimates. Observed survival is an estimate of the probability of surviving all causes of death [24]. Relative survival is defined as the ratio of the proportion of observed survivors (all causes of death) in a cohort of cancer patients to the proportion of expected survivors in a comparable cohort of cancer-free individuals, assuming independent competing causes of death. Since a cohort of cancer-free individuals is difficult to obtain, SEER uses expected life tables and assumes cancer deaths are a negligible proportion of all deaths [24].

## 3. Results

The median ages of CMM, MCC and MM patients were 62, 75, and 66 years, respectively. Analysis showed that 58%, 63% and 36% of the cases of CMM, MCC and MM, respectively, occurred in males. The incidence of MCC in this population was 5.9 per 1,000,000 individuals in 2000–2019, while the incidence of MM was only 0.1 per million. However, the rates of all three cancers increased from 2000–2009 to 2010–2019, with a tripling in the rate of MM cases.

These cancers are distinguished by the locations of their associated tumors in the body. In the case of CMM, 100% of the tumors occur on the skin, while 98% of MCC tumors occur in the non-epithelial skin (Table 1). CMM is found only on the skin, but the tumors can be located anywhere on the external skin. MM tumors are found on the same body sites as MCC, apart from the breasts. The data showed that MMs account for less than 0.060% of all melanomas. MCC was 25–80 times more common than MM, while CMM was 30–40 times more common than MCC. Neither primary MM nor MCC cancers were found in the brain nor other parts of the nervous system, endocrine system, or blood. MCCs also occur in the oral cavity, pharynx, and other miscellaneous sites, as well as in soft tissues, including the heart and respiratory system. MMs by definition occur in the inner lining of the mucosa and not in the skin, hence it is not clear whether those reported on the skin are actually mucosal melanomas of unknown primary or whether they are melanomas of the skin. Most MMs in men occur in the respiratory system, followed by the oral cavity and pharynx, the skin, and finally the digestive system (Table 1). Among women, MMs occur mostly in the genital system, followed by the respiratory system, the oral cavity and pharynx, and finally the skin and digestive system.

**Figure 1:**
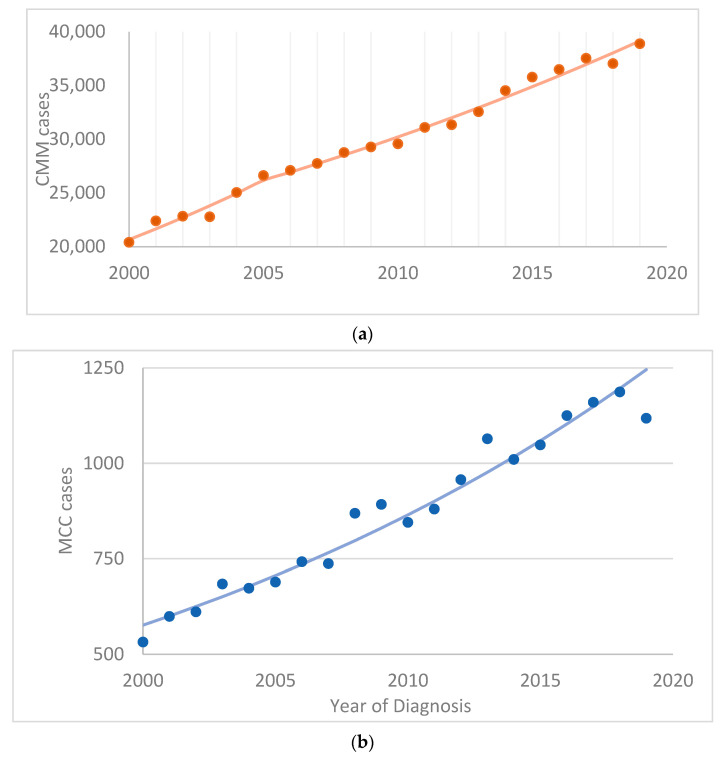

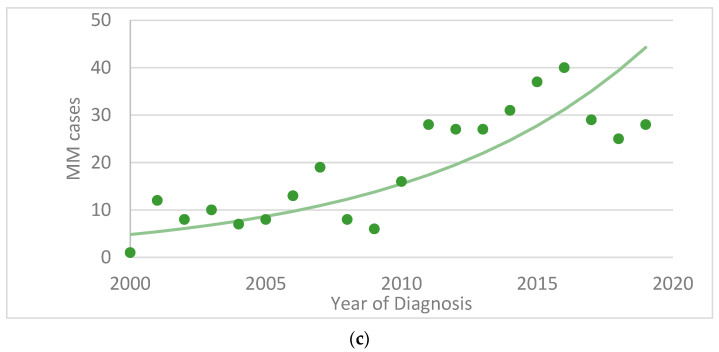
Number of cases and APC-modeled regression line graphed over time for (**a**) cutaneous malignant melanoma (CMM), (**b**) Merkel cell carcinoma (MCC) and (**c**) mucosal melanoma (MM).

Figure 1 shows the number of CMM, MCC and MM cases over time. The cases are presented separately due to the large differences in the numbers. More variation is observed for MM, likely due to the small number of cases, with higher percentages of cases reported starting from 2011. Figure 1 also shows a steady increase in the number of cases of all three cancer types from 2000 to 2019.

Table 2 reports the populations from the 16 states present in the 22 SEER registries based on the 2020 census and the distribution by state as a percentage of all SEER (expected percentage of the total). The table also presents the percentage of CMM, MCC and MM cases in each registry by state. Similar percentages of CMM and MCC cases were seen in each state. The largest population in the registry is from California, followed by Texas and New York. California had more CMM and MCC cases, but fewer MM cases, than expected based on the population size. Conversely, a higher than expected percentage of MM cases based on population size was seen in Texas and New York. These differences reflect a difference in the etiology of MM compared with the etiology of CMM and MCC.

**Figure 2:**
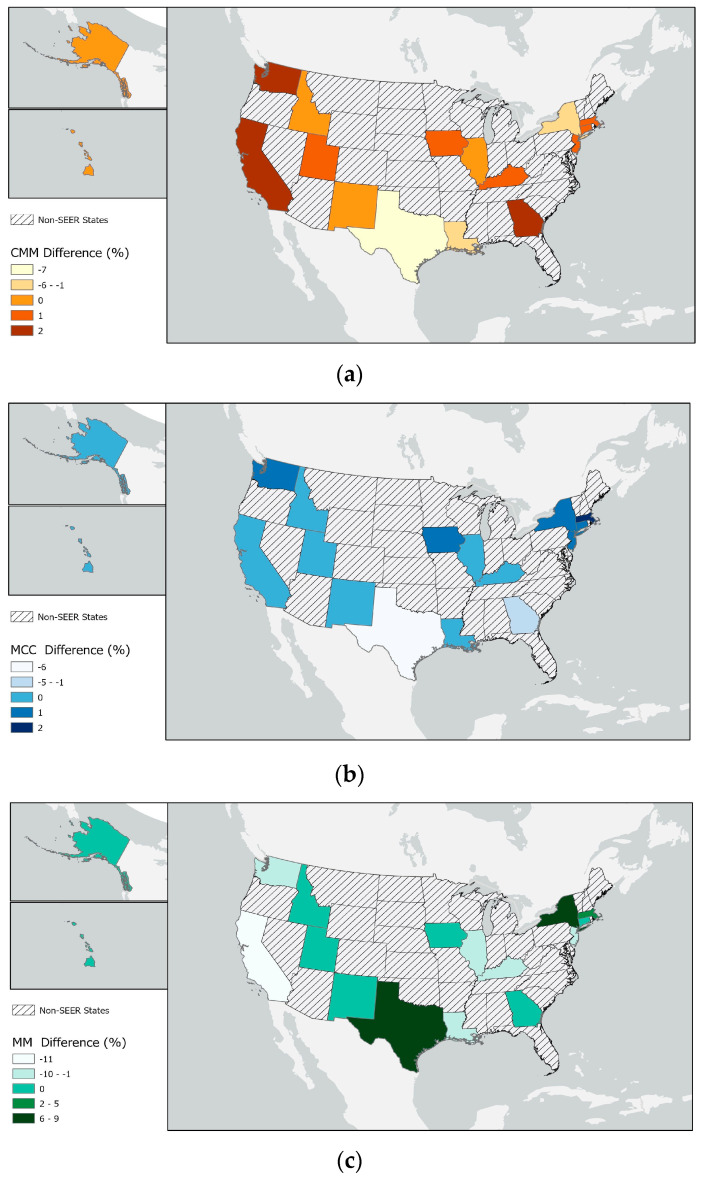
Differences in the percentages of expected (**a**) cutaneous malignant melanoma (CMM), (**b**) Merkel cell carcinoma (MCC) and (**c**) mucosal melanoma (MM) cases in the 16 states in the 22 cancer registries in SEER 2000–2019 were mapped.

The differences in the numbers of observed and expected cancer cases in each state are mapped in Figure 2. We aggregated the data from each state; however, only Alaskan Natives and the 13 counties that make up Puget Sound are in the Alaska and Washington state datasets, respectively.

Table 3 shows the age-adjusted incidence rates of these cancers in the 22 registries. MCC is 32 times less common than CMM in men and 38 times less common than CMM in women based on the age-adjusted incidence rates. CMM and MCC rates increase sharply with age, particularly among men; MM rates also increase with age (Table 3). The overall incidence of CMM was 203.7 per 1,000,000 people in 2000–2019, with higher rates noted among men. Women had 70% of the CMM incidence rate of men. The rarer MCC had an incidence of 5.9 per 1,000,000 people, with the rates also being higher in men; women had 58% of the incidence rate of men. The overall incidence of MM was 0.1 per 1,000,000 people, with women having 175% the incidence rate of men. For these cancers, the incidence was highest in non-Hispanic Whites, followed by American Indian/Alaskan Natives and Hispanics; the lowest incidence was in non-Hispanic Blacks (Table 3). Stage varied by cancer type, as seen in Table 3. A higher proportion of MM and MCC cases were diagnosed at distant stage compared with CMM cases. One third of CMM and MCC cases were unstaged, while 28% of MM cases were unstaged (i.e., blank, unknown or unstaged). For most patients—79% with CMM, 71% with MCC and 80% with MM—the data represents the first primary cancer diagnosis (Table 3).

Five-year overall relative survival was high among patients with CMM, but lower among patients with MCC and MM (Table 4). For all three cancers, survival was highest in younger individuals and lowest in individuals aged 75+ years. Females with CMM and MCC had slightly higher survival, but slightly lower survival with MM. As expected, survival was best among individuals with localized stage and worst among individuals with distant stage disease. Individuals with unstaged CMM and MCC seemed to have survival rates similar to the average of the other stages, whereas for MM, the highest survival rate was noted among individuals with unstaged cancer. Survival in individuals with CMM was lowest among non-Hispanic Blacks, whereas Hispanics with MM had the lowest survival. However, the lowest MCC survival rate was noted among non-Hispanic Black males and non-Hispanic American Indian/Alaskan Native females. We attempted to analyze Breslow tumor thickness to better understand unstaged cancer but found a large percentage of missing data, with all tumor thicknesses data for MCC cases reported as unknown.

**Figure 3:**
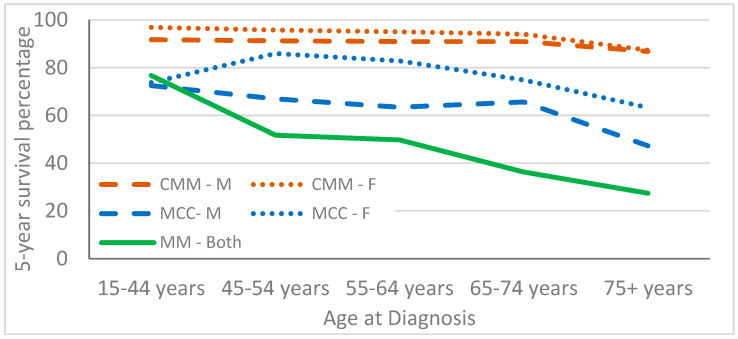
Relative survival by age for people with cutaneous malignant melanoma (CMM), Merkel cell carcinoma (MCC) and mucosal melanoma (MM) in 17 cancer registries in SEER 2000–2019.

Figure 3 shows the relative survival of individuals with the three types of cancers by age and sex. CMM has the highest survival rate in both males and females, followed by MCC; both had higher survival rates in females. Due to the small number of MM cases, we plotted the combined male and female survival rates. The MM survival rate was 77% in individuals aged 15–44, but this dropped to 27% in people aged 75 years and older.

## 4. Discussion

Several similarities were observed among CMM, MCC and MM, including increasing incidence rates with age and increasing number of cases in more recent diagnosis years. Overall, similar proportions of cases to population numbers were observed for CMM and MCC across the 22 SEER registries; however, the percentage of cases in Texas was lower than expected based on the population size. MM was more prevalent than expected based on the population size in Texas and New York but was only half of that expected in California. Finding more CMM and MCC cases than expected by population size in California is likely due to the increased opportunities for UVR exposure in this location. However, the reasons for the higher than expected rates of MM in Texas and New York and the lower than expected rates in California based on population size are unclear. While there are research centers with MM experts in Texas and New York, SEER registries only report cases of patients who are resident in their catchment areas. Understanding the reasons for the higher number of cases in Texas and New York might be important for unravelling the etiology of MM.

The National Cancer Institute (NCI) defines rare cancers as those that affect fewer than 40,000 people per year in the US [28]; both MCC and MM fit this definition. Rare cancers are challenging for patients, doctors, and researchers due to limited information. The higher survival among younger cancer patients is mainly due to diagnosis at earlier stages of the disease [29]. The lower survival rates among patients with MCC and MM (SEER-17) increase the need to better understand the risk factors for these cancers.

Fewer MCC cases were diagnosed at the localized stage compared with CMM cases. MM had an even higher percentage of later stage diagnosis, specifically distant stage cancers, compared with MCC. This suggests that there are additional factors affecting the diagnosis of MCC and MM compared with CMM. The descriptive analyses presented here form the initial phase of elucidating the etiology of these cancers. Each cancer is discussed below; most of the discussion is focused on MCC and MM since less is known about their etiology compared with CMM.

### 4.1. Cutaneous Malignant Melanoma (CMM)

Since cumulative UVR increases as we age, CMM rates also increase with age [30], although it can also be diagnosed in children, adolescents and young adults. Early age at first use of tanning beds has been linked to melanoma and is thought to be associated with the increased incidence of the disease among women under the age of 40 years [31]. Immunosuppressed patients are at increased risk of developing CMM but are at a reduced risk of developing MCC [32].

The main symptoms of CMM are the development of a new mole or a change in the appearance of a mole, including changes in shape (asymmetry, uneven or ragged borders), color or size (>6 mm in diameter) and bleeding, becoming crusty, itchy, or sore. Therefore, screening for CMM includes self-skin examination [33] and a clinical skin exam, preferably by a dermatologist, to check suspicious lesions. Regardless of skin tone, the development of lesions or changes in skin lesions should be evaluated. Screening can include the use of a dermoscopy, reflectance confocal microscopy and other imaging techniques, when available, to identify suspicious lesions [33]. If suspected to be cancerous, the lesion is biopsied and sent to a pathology laboratory for histologic examination. People at high risk of developing skin cancer may also conduct skin self-examinations or have a partner check their skin and then go to a dermatologist if a suspicious lesion is found. The American Cancer Society provides a link to “how to do a skin self-exam” (https://www.cancer.org/healthy/be-safe-in-sun/skin-exams.html (accessed on 28 February 23)). Surgery is the primary treatment for melanoma, although immunotherapies have been developed for metastatic melanoma [34].

### 4.2. Merkel Cell Carcinoma (MCC)

Apart from the skin (98% of MCC), 1% of MCCs occurred in the oral cavity and pharynx, including 27% on the lip and 66% in the salivary glands. Additionally, one study reported that CMM is associated with a three-fold greater risk of MCC [35]. MCC is potentially associated with UVR exposure, explaining some of the increased risk that comes with increasing age, as seen for CMM [1,4,5,6,15,17,36], and the increase with age seen in this study (Table 2). The incidence of MCC in the UK, which ranges from 0.1 to 0.2 per 100,000 people [37], is slightly lower than that observed here, i.e., 0.4 to 0.8 (4.4 to 7.6 per 1,000,000 people). One study reported that based on the US National Cancer Database, approximately 58–74% of patients present with localized disease, 23–52% present with regional stage, and 2–3% present with metastasis at the time of diagnosis [38]. The SEER-22 cancer registries contain 40% localized cancers and 18% regional cancers; however, 34% of MCCs were reported as unstaged. We observed higher rates of both CMM and MCC in males. Another study also reported this sex difference and found that MCC and CMM are distributed across similar body sites [39].

The increase in the incidence of MCC since 2000 is thought to be due to the advancing age of the population, UVR triggers, and an increasing number of immunocompromised individuals in the US [18]. Merkel cell polyomavirus has been identified in 80–85% of MCC cases in the northern hemisphere [1,2,4,5,17,40,41,42,43]. However, Merkel cell polyomavirus cases also increase with age in the general population, for example from 50% in children to 80% in adults older than 50 years in Belgium and Italy [36,44]. Merkel cell polyomavirus infections can persist for life and this is the only polyomavirus associated with human cancers. This polyomavirus is believed to be the leading cause of MCC [14,45,46]. Merkel cell polyomavirus is common both in patients with MCC and in the general population.

MCC is also strongly associated with prior transplantation [47], HIV [48,49], other viruses [42] and general immunocompromised systems [18,50]. It is not surprising that those who are immunocompromised have a 5–50-fold increased risk of developing MCC (10% of cases) given the viral etiology [5,32,51]. MCC has also been detected following immunosuppressive therapy [52,53,54,55]. Those on immunosuppressive therapy following organ transplants or those who are immunocompromised due to HIV infections have an increased risk of developing MCC that follows a path different from that of those exposed to high UVR or those who are infected with Merkel cell polyomavirus [56]. While CMM also shows an association with immunocompetence, MCC is 2–10 times more likely to develop following transplantation or after HIV infection [32]. Furthermore, MCC has been associated with blood diseases [57]. Blood diseases have had poor outcomes with COVID-19 infections, hence MCC may as well [58,59]. Only case reports (without control subjects) have suggested a potential association between MCC and autoimmune disorders [5]. This evidence shows the need for further investigation into the association between MCC and other viruses.

Similar to CMM, screening for MCC can be conducted via clinical examination for unusual growths; a biopsy of suspicious skin is taken and sent to a pathology laboratory for histologic examination. At a population level, people are less aware of MCC and therefore are less likely to request a skin exam specifically in relation to this cancer. MCC can grow rapidly and has a high risk of local recurrence and metastasis at an early stage. A third of patients have regional lymph-node involvement and one in 10 patients has distant metastases at the time of diagnosis [14,60,61]. Chemotherapy is not indicated for the primary treatment of MCC. Radiating the affected area after local excision and sentinel lymph node biopsy should be considered. Advances in immunotherapies provide an alternative to chemotherapies [17]. Choices around chemotherapy for patients who are not candidates for immunotherapy due to immune-suppressive therapy, active HIV, HBV, or HCV infections depend on the oncologist guiding the MCC management plan [56].

### 4.3. Mucosal Melanoma (MM)

Analysis of MM cases in 2000–2019 showed that 17% of the cases occurred in the oral cavity and pharynx, 76% occurred in the mouth and gums, 30% occurred in the respiratory system, 22% in the female genital system, 17% in the oral cavity and pharynx, 11% in the digestive system, 4% in the eye and eye orbit, 2% in the urinary system and 1% in the male genital system. A 1985–1994 study of the National Cancer Database, a cancer registry of 1227 hospitals, reported 1074 MM cases, with 55.4% of them occurring in the head and neck, 18% in the female genital tract, 23.8% in the anal/rectal region and 2.8% in the urinary tract [62]. A study of 30 MM patients aged 32–85 were diagnosed with head and neck mucosal melanomas (oral cavity and pharynx) at Emory Hospital between 1986 and 2006. Sinonasal melanomas were more common than oral melanomas [63]. Similarly, a previous study of the SEER 1987–2009 registries reported that 73% of head and neck MM cases were sinonasal [64]. A population-based study of MM in southern Europe reported that 29% of the cases occurred in the head and neck, 36% were rectal cases, 33% occurred in the female genitals and 2% were urethral cases, with equal or higher numbers among females [7]. Of 46 MM cases reported in a US study in Boston, MA, from 2011 to 2019, 41% occurred in the female genital system, 35% were anal/rectal cases, and 22% occurred in the sinus/nasopharynx [65]. These cases are likely included in the SEER data presented here.

The high proportion of unstaged MM cases in the SEER program is due to the difficulty in staging MM. Although there are NCCN staging guidelines for head and neck mucosal melanomas, staging for MMs originating in other sites are adapted from CMM and cancers in similar sites [60,66]. The lymph node basin draining the cancer site should be evaluated, which in the case of CMM or MCC, is via a sentinel lymph node biopsy. However, for MM originating in the head and neck, rectum, anal cavity or genital-urinary system, a sentinel lymph node biopsy may not be possible and the regional lymph node basin may only be radiologically evaluated. Further, as MMs typically occur in non-visualized areas, diagnosis occurs, by default, at a later time point compared with surface skin lesions.

Similar to our results, several other MM studies reported younger age as an important prognostic factor [23,67,68,69,70]. MM rates seemed to be higher in Asian populations [23,71]. In the US, the median age at diagnosis prior to 1995 was approximately 70 years [72]; however, we saw a decrease in the median age to 66 years. A large study in China reported a mean age of 55 years and found that 65% of the patients were female [73]. They reported higher survival in individuals under the age of 40 years, lower depth of invasion, no positive nodes or distant metastases, and respiratory and head and neck anatomic sites for MM. Furthermore, they suggested higher survival in individuals with MM arising in the respiratory tract compared with those with MM arising in the head and neck [73]. Similar to our study, they also found that individuals, especially females, diagnosed at early stages of the disease had better survival outcomes compared with individuals diagnosed at a late stage [73]. MMs have high rates of recurrence following initial surgery [65], making understanding their etiology even more important.

MMs are so rare that comprehensive analysis of their risk factors has not been conducted, and what is known about them comes primarily from case reports. Here we described risk factors in a larger population using 20 years of SEER registry data. However, large studies with controls are needed to compare risk factors.

Similar to CMM and MCC, MM can be detected during a physical exam and confirmed by a biopsy. However, MM hides in places we cannot easily see. Those at risk are unlikely to know they are at risk and so are less likely to get regular skin exams, leading to diagnosis at later stages of the disease. Oral MM may be detected by a dentist noticing an ulcer or discoloration; vaginal MM may be found on a routine Pap test; and anal MM may be found with a digital rectal exam or anal pap test. Recurrence is common. Access to care and SES may play a role but were not examined in this study. Reviewing the literature showed that therapeutic protocols with the best survival seemed to use a combination of radiation therapy, chemotherapy, and targeted therapy or definitive proton beam radiation therapy [67]. Adequate clinical trials to compare therapies are lacking due to limited number of patients.

## 5. Conclusions

MCCs are 45 times more common in both sexes compared with MM, while CMMs are 35 times more common than MCCs. The three cancers have different distributions across the body. Like other cancers, the incidences of these three cancers increase with age and are associated with poorer survival with increasing age at diagnosis and later stage at diagnosis. While CMM survival is lowest among non-Hispanic Blacks, MM survival is lowest in Hispanics. MM accounts for only 0.060% of all melanomas. Although the rates of MM reported here were lower than those reported in other studies, we observed a tripling of the number of cases from 2000-09 to 2010-19. The percentage of MM cases in the SEER registries differ from expected based on census data; they also differ from the percentages of CMM and MCC.

More research is needed to understand the etiologies of MCC and MM as they are rare and, thus, understudied.

## Figures and Tables

**Table 1:** Locations of malignant melanomas and Merkel cell carcinoma on the bodies of patients in 22 cancer registries in SEER 2000–2019.

Body Location		CMM			MCC			MM	
	Male	Female	All	Male	Female	All	Male	Female	All
All Skin surfaces *	347,752	249,989	597,741	10,686	6387	17,073	25	28	53
Melanoma of the skin	347,752	249,989	597,741	0	0	0	25	28	53
Other non-epithelial skin	0	0	0	10,686	6387	17,073	0	0	0
**All Sites**	347,752	249,989	597,741	10,918	6504	17,422	138	242	380
**Sites other than skin**	0	0	0	232	117	349	113	214	327
Oral cavity and pharynx	0	0	0	111	35	146	32	31	63
Digestive system	0	0	0	3	2	5	15	27	42
Respiratory system	0	0	0	12	22	34	49	65	114
Bones and joints	0	0	0	0	0	0	0	0	0
Soft tissue, including heart	0	0	0	29	19	48	0	0	0
Breasts	0	0	0	2	0	2	0	0	0
Female genital system	0	0	0	0	12	12	0	82	82
Male genital system	0	0	0	3	0	3	3	0	3
Urinary system	0	0	0	1	0	1	3	3	6
Eye and eye orbit	0	0	0	4	4	8	10	6	16
Brain and other nervous systems	0	0	0	0	0	0	0	0	0
Endocrine system	0	0	0	1	0	1	0	0	0
Blood *	0	0	0	0	0	0	0	0	0
Miscellaneous	0	0	0	66	23	89	1	0	1

CMM, cutaneous malignant melanoma; MCC, Merkel cell carcinoma; MM, mucosal melanoma. * Blood cancers include lymphoma, myeloma and leukemia.

**Table 2:** Percentages of cutaneous malignant melanoma (CMM), Merkel cell carcinoma (MCC) and mucosal melanoma (MM) in 16 states represented in 22 cancer registries in SEER 2000–2019.

SEER Registry	2020	% of Total	CMM			MCC			MM		
	Census	(Expected)	Male	Female	All	Male	Female	All	Male	Female	All
Alaska Natives	111,575	**0%**	0%	0%	0%	0%	0%	0%	1%	0%	0%
California (4 SEER sites)	39,538,223	**25%**	28%	25%	27%	26%	25%	25%	12%	14%	14%
Connecticut	3,605,944	**2%**	3%	3%	3%	3%	4%	3%	2%	2%	2%
Georgia (3 SEER sites)	10,711,908	**6%**	8%	8%	8%	5%	4%	5%	3%	7%	6%
Hawaii	1,455,271	**1%**	1%	1%	1%	1%	1%	1%	2%	2%	2%
Iowa	3,190,369	**2%**	3%	3%	3%	3%	3%	3%	1%	2%	2%
Idaho	1,839,106	**1%**	1%	1%	1%	1%	1%	1%	1%	0%	1%
Illinois	12,812,508	**8%**	8%	8%	8%	7%	8%	8%	7%	5%	6%
Kentucky	4,505,836	**3%**	4%	4%	4%	3%	3%	3%	2%	1%	2%
Louisiana	4,657,757	**3%**	3%	2%	2%	3%	3%	3%	1%	2%	2%
Massachusetts	7,029,917	**4%**	5%	6%	5%	6%	7%	6%	6%	11%	9%
New Jersey	9,288,994	**6%**	7%	7%	7%	7%	8%	7%	4%	5%	5%
New Mexico	2,117,522	**1%**	1%	1%	1%	1%	1%	1%	2%	2%	2%
New York	20,201,249	**13%**	12%	12%	12%	13%	16%	14%	15%	22%	20%
Texas	29,145,505	**18%**	11%	10%	11%	13%	11%	12%	36%	22%	27%
Utah	3,271,616	**2%**	3%	3%	3%	2%	1%	2%	2%	1%	2%
Washington (Puget Sound)	5,301,675	**3%**	4%	5%	5%	4%	4%	4%	1%	0%	1%

CMM, cutaneous malignant melanoma; MCC, Merkel cell carcinoma; MM, mucosal melanoma.

**Table 3:** Demographics and rates * of cutaneous malignant melanoma (CMM), Merkel cell carcinoma (MCC) and mucosal melanoma (MM) in 22 cancer registries in SEER 2000–2019.

Demographics		CMM			MCC			MM	
	Male	Female	All	Male	Female	All	Male	Female	All
**Overall Rate in 2000–2019 per 1,000,000**	240.6	167.8	203.7	7.6	4.4	5.9	0.10	0.16	0.13
**Rate in 2000–2009**	209.7	151	179.9	6.2	3.8	5	0.05	0.08	0.07
**Rate in 2010–2019**	269.1	183.3	225.6	8.8	4.8	6.8	0.14	0.24	0.19
% 1st primary malignant cancer	76%	83%	79%	68%	75%	71%	79%	80%	80%
**Age**									
15–44 years	59.9	94.1	76.8	0.2	0.1	0.2	0.0	0.0	0.0
45–54 years	247.0	225.5	236.1	2.1	1.2	1.7	0.1	0.1	0.1
55–64 years	504.8	299.2	398.2	8.8	4.8	6.7	0.2	0.4	0.3
65–74 years	933.3	404.9	648.3	31.0	13.7	21.7	0.4	0.5	0.4
75+ years	1466.2	504.3	879.1	94.0	38.0	59.8	0.6	0.8	0.7
Race/Ethnicity *									
Non-Hispanic White	399.4	268.5	332.9	12.3	6.7	9.5	0.1	0.2	0.2
Non-Hispanic Black	7.7	9.0	8.4	0.8	0.7	0.7	0.1	0.0	0.1
Non-Hispanic American Indian/Alaska Native	62.0	55.0	58.5	3.1	2.1	2.6	0.2	0.0	0.1
Non-Hispanic Asian or Pacific Islander	13.3	12.2	12.8	1.4	1.1	1.2	0.1	0.1	0.1
Hispanic (All Races)	25.0	31.7	28.3	1.5	1.7	1.6	0.0	0.1	0.1
Non-Hispanic Unknown Race	~	~	~	~	~	~	~	~	~
**Percentage at each Stage**									
Localized	56%	58%	57%	39%	43%	40%	26%	37%	33%
Regional	7%	6%	7%	19%	16%	18%	30%	28%	28%
Distant	4%	3%	3%	8%	6%	7%	14%	10%	11%
Unstaged	6%	6%	6%	8%	8%	8%	6%	5%	6%
Blank(s)	27%	28%	27%	27%	28%	27%	25%	21%	22%

CMM, cutaneous malignant melanoma; MCC, Merkel cell carcinoma; MM, mucosal melanoma. * Rates are per 1,000,000 people.

**Table 4:** Five-year survival and Breslow thickness of cutaneous malignant melanoma (CMM), Merkel cell carcinoma (MCC) and mucosal melanoma (MM) cases in 17 cancer registries in SEER 2000–2019.

5-Year Survival Percentage	CMM				MCC				MM			
	Male		Female		Male		Female		Male		Female	
	Obs.	Rel.	Obs.	Rel.	Obs.	Rel.	Obs.	Rel.	Obs.	Rel.	Obs.	Rel.
Overall	79.4	90.4	87.3	94.4	43.5	57.0	54.5	70.5	47.3	50.2	41.2	45.3
									**Males & Females**			
**Age**										**Obs.**	**Rel.**	
15–44 years	90.7	91.7	96.4	96.9	71.7	72.5	73.1	73.7		76.2	76.7	
45–54 years	88.5	91.2	94.0	95.7	65.0	66.9	84.9	85.9		50.7	51.7	
55–64 years	85.5	90.9	91.6	95.0	59.5	63.4	79.6	82.8		48.0	49.7	
65–74 years	79.5	90.9	85.9	94.0	57.2	65.6	68.0	74.8		33.3	36.3	
75+ years	54.3	86.8	59.1	87.3	28.4	47.3	40.3	63.3		20.2	27.4	
**Stage**												
Localized	86.3	98.1	92.1	99.3	49.0	66.5	60.5	78.9		65.6	72.1	
Regional	54.8	62.3	62.9	69.9	45.5	57.3	51.9	65.7		26.9	28.8	
Distant	19.1	21.6	22.5	24.8	14.1	18.4	28.0	33.4		9.1	9.4	
Unstaged	69.9	81.2	78.3	86.0	38.1	49.6	43.8	62.7		83.3	86.4	
Breslow Thickness *	**Male**		**Female**		**Male**		**Female**		**Male**		**Female**	
≤0.1 mm	2.9%		2.7%		0.0%		0.0%		0.0%		1.0%	
0.2–9.7 mm	49.1%		48.5%		0.0%		0.0%		8.5%		17.3%	
9.8+ mm	0.9%		0.7%		0.0%		0.0%		0.0%		3.1%	
Unknown	47.2%		48.1%		100.0%		100.0%		91.5%		78.6%	

CMM, cutaneous malignant melanoma; MCC, Merkel cell carcinoma; MM, mucosal melanoma. Actuarial method used by SEER*Stat for SEER 17 registries excluded subjects diagnosed <5 years prior to submission. * Breslow thickness is a measure of melanoma thickness.

## Data Availability

Data presented in this study are openly available through https://seer.cancer.gov/data/ and can be obtained directly from SEER with an agreement.
